# Transformer Models, Graph Networks, and Generative AI in Gut Microbiome Research: A Narrative Review

**DOI:** 10.3390/bioengineering13020144

**Published:** 2026-01-27

**Authors:** Yan Zhu, Yiteng Tang, Xin Qi, Xiong Zhu

**Affiliations:** 1 Department of Ophthalmology, Scheie Eye Institute, University of Pennsylvania, Philadelphia, PA 19104, USA; 2 NanoScience Technology Center, University of Central Florida, Orlando, FL 32816, USA; yiteng.tang@ucf.edu; 3 Neurosurgery Research Laboratory, West China Hospital, Sichuan University, Chengdu 610041, China; qixin72@126.com; 4 Department of Prenatal Diagnosis, Chengdu Women’s and Children’s Central Hospital, School of Medicine, University of Electronic Science and Technology of China, Chengdu 611731, China

**Keywords:** metagenomics, transformer models, multi-modal AI, artificial intelligence, gut microbiome, machine learning, personalized medicine, multi-omics integration

## Abstract

Background: The rapid advancement in artificial intelligence (AI) has fundamentally reshaped gut microbiome research by enabling high-resolution analysis of complex, high-dimensional microbial communities and their functional interactions with the human host. Objective: This narrative review aims to synthesize recent methodological advances in AI-driven gut microbiome research and to evaluate their translational relevance for therapeutic optimization, personalized nutrition, and precision medicine. Methods: A narrative literature review was conducted using PubMed, Google Scholar, Web of Science, and IEEE Xplore, focusing on peer-reviewed studies published between approximately 2015 and early 2025. Representative articles were selected based on relevance to AI methodologies applied to gut microbiome analysis, including machine learning, deep learning, transformer-based models, graph neural networks, generative AI, and multi-omics integration frameworks. Additional seminal studies were identified through manual screening of reference lists. Results: The reviewed literature demonstrates that AI enables robust identification of diagnostic microbial signatures, prediction of individual responses to microbiome-targeted therapies, and design of personalized nutritional and pharmacological interventions using in silico simulations and digital twin models. AI-driven multi-omics integration—encompassing metagenomics, metatranscriptomics, metabolomics, proteomics, and clinical data—has improved functional interpretation of host–microbiome interactions and enhanced predictive performance across diverse disease contexts. For example, AI-guided personalized nutrition models have achieved AUC exceeding 0.8 for predicting postprandial glycemic responses, while community-scale metabolic modeling frameworks have accurately forecast individualized short-chain fatty acid production. Conclusions: Despite substantial progress, key challenges remain, including data heterogeneity, limited model interpretability, population bias, and barriers to clinical deployment. Future research should prioritize standardized data pipelines, explainable and privacy-preserving AI frameworks, and broader population representation. Collectively, these advances position AI as a cornerstone technology for translating gut microbiome data into actionable insights for diagnostics, therapeutics, and precision nutrition.

## 1. Introduction

The human gut microbiome comprises a complex and dynamic ecosystem of trillions of microorganisms that reside primarily in the large intestine [1]. These microbes—bacteria, archaea, viruses, and fungi—collectively encode over 100 times more genes than the human genome and contribute essential metabolic, immunological, and neurological functions to the host [2]. A well-balanced microbiome supports digestion, nutrient absorption, immune modulation, and gut-brain communication, playing a central role in maintaining human health [3,4,5]. Conversely, disruptions in microbiome composition, often referred to as dysbiosis, have been implicated in a wide array of diseases, including inflammatory bowel disease, obesity, diabetes, cancer, and neurodegenerative disorders [6,7]. Together, these functions underscore the central role of the gut microbiome in maintaining host physiological homeostasis.

Over the past two decades, advances in high-throughput sequencing and computational biology have catalyzed a wave of microbiome research aimed at decoding the functional roles of gut microbes and their interactions with the host [8,9,10]. However, as microbiome datasets have grown in volume and complexity—with thousands of microbial features per sample across diverse populations and longitudinal designs—traditional statistical tools have proven inadequate to fully interpret these data [9,11]. Similar challenges of high-dimensional and longitudinal phenotypic data integration have been extensively discussed in AI-based biomedical research, including ophthalmology and complex genetic diseases [12,13]. There is thus a growing need for more sophisticated analytical frameworks that can capture nonlinear relationships, integrate heterogeneous data types, and identify subtle but biologically meaningful patterns across large-scale microbiome datasets.

Artificial intelligence (AI), particularly machine learning (ML) and deep learning (DL), has emerged as a transformative force in microbiome research, offering powerful methods to analyze high-dimensional, sparse, and heterogeneous data [14,15,16]. Machine learning algorithms are capable of discovering hidden structure in complex datasets without pre-specified hypotheses, making them ideal for applications ranging from microbial signature discovery to disease classification and treatment response prediction [17,18]. In contrast to traditional models that rely on linear assumptions or fixed statistical tests, AI models can learn intricate nonlinear associations and interactively adapt as more data becomes available [14,15,16].

AI methodologies for microbiome data analysis have expanded rapidly in recent years, ranging from classical machine learning approaches to deep learning, graph-based models, and transformer-based architectures [19,20,21]. These tools are being used not only to predict disease states but also to simulate microbial community dynamics, optimize dietary interventions, and integrate multi-omics information. Beyond computational advances, emerging medical biosensing technologies may further expand AI-driven microbiome research by enabling higher-resolution and real-time data acquisition [22,23]. Meanwhile, there is increasing emphasis on explainability, standardization, and clinical interpretability—ensuring that AI-driven insights can be translated into meaningful applications in personalized medicine and microbiome-based therapies [14,24].

**Figure 1:**
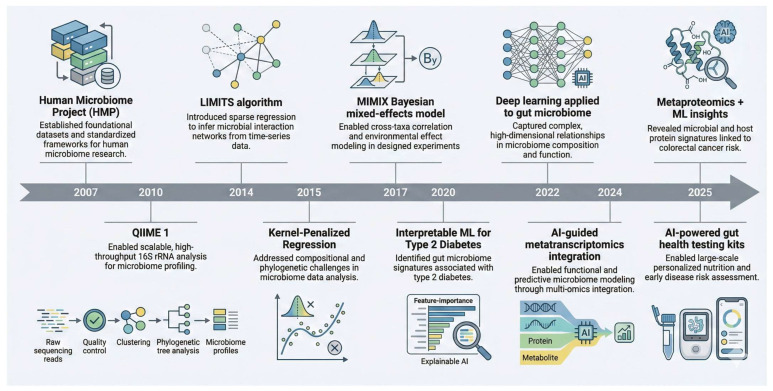
Milestones in AI-driven gut microbiome research.

The aim of this narrative review is to systematically synthesize recent advances in AI methodologies applied to gut microbiome research, critically evaluate their roles in therapeutic optimization, personalized nutrition, and precision medicine, and identify current limitations and future directions for clinical translation. Figure 1 summarizes key milestones in the integration of artificial intelligence (AI) into gut microbiome research over the past two decades. The Human Microbiome Project (HMP, 2007) provided foundational datasets, standardized protocols, and analytic frameworks that catalyzed microbiome science [25]. The release of QIIME 1 in 2010 enabled high-throughput analysis of 16S rRNA sequencing data, marking a pivotal advance in microbiome multi-omics processing [26]. Subsequent statistical innovations, such as the LIMITS algorithm (2014) for inferring microbial interaction networks, Kernel-Penalized Regression (2015), and the MIMIX Bayesian mixed-effects model (2017), laid the groundwork for more sophisticated modeling of ecological and experimental data. The application of interpretable machine learning for type 2 diabetes biomarker discovery in 2020 demonstrated the translational potential of AI-microbiome approaches [27]. In 2022, deep learning frameworks were highlighted for their capacity to capture complex, high-dimensional microbial relationships [28]. Recent advances have focused on integrating multi-omics layers, as exemplified by AI-guided metatranscriptomics integration (2024) and metaproteomics-based machine learning models for colorectal cancer risk prediction (2025) [29,30]. Concurrently, commercial translation has accelerated, with AI-powered consumer gut health testing kits, such as those developed by Viome, achieving widespread adoption by 2025 [31].

### Review Methodology and Scope

This article is a narrative review intended to provide a structured and critical overview of recent advances in AI applications for gut microbiome research, with particular emphasis on transformer-based models, graph neural networks, generative AI, and multi-modal learning frameworks.

The literature was identified through non-systematic searches of major scientific databases, including PubMed, Google Scholar, Web of Science, and IEEE Xplore. Searches were conducted using combinations of keywords such as *gut microbiome*, *artificial intelligence*, *machine learning*, *deep learning*, *transformer models*, *graph neural networks*, *multi-omics integration*, and *digital twins*. The primary focus was placed on peer-reviewed articles published between approximately 2015 and early 2025, capturing both foundational studies and recent methodological advances.

Inclusion criteria comprised studies that (i) applied machine learning or deep learning methods to human gut microbiome data, (ii) reported quantitative predictive, modeling, or simulation outcomes relevant to diagnostics, therapeutic optimization, or personalized nutrition, and (iii) introduced methodological innovations or engineering frameworks with potential translational relevance. Exclusion criteria included studies focused exclusively on animal models without clear methodological transferability, purely experimental microbiome studies without AI-based analysis, and opinion or commentary articles lacking technical or empirical contributions.

When multiple publications analyzed overlapping datasets (e.g., Human Microbiome Project or American Gut cohorts), priority was given to studies demonstrating greater methodological novelty, larger or longitudinal sample sizes, external validation, or improved interpretability. Although no formal risk-of-bias scoring system was applied, a qualitative assessment of study strength was performed during synthesis, privileging studies with transparent model evaluation, cross-cohort validation, multi-omics integration, or mechanistic interpretability over purely descriptive or single-cohort analyses.

As a narrative review, this work does not aim to exhaustively capture all published studies or to provide a quantitative meta-analysis. Instead, it seeks to organize representative and influential contributions into a coherent engineering-focused framework, highlighting comparative methodological trade-offs, emerging design principles, and high-priority challenges for the development and clinical translation of AI-driven gut microbiome technologies.

## 2. Therapeutic Optimization via Microbiome Simulation

How can AI-based models be designed to robustly predict and optimize microbiome-targeted interventions across heterogeneous individuals and dynamic microbial ecosystems, while balancing mechanistic interpretability, scalability, and clinical feasibility? The human gut microbiome represents both a therapeutic target and a potential delivery system for novel interventions aimed at restoring host health, with approaches such as probiotics, prebiotics, and fecal microbiota transplantation (FMT) demonstrating potential in clinical settings [32]. However, the complexity and individual specificity of microbial ecosystems pose major barriers to universal therapeutic efficacy. For example, the success of FMT, probiotics, and prebiotics typically varies across individuals due to differences in microbial community composition, host genetics, diet, and environmental exposures, frequently leading to inconsistent or transient outcomes [32]. In response, in silico simulation frameworks that apply AI for predictive modeling of therapeutic interventions are emerging as powerful strategies to manage heterogeneity. These computational approaches enable researchers to explore how interventions may reconfigure the gut ecosystem and influence host physiology before clinical deployment [33]. By forecasting individual responses and ecosystem-wide outcomes, AI-driven simulations offer a promising path toward the design of personalized and effective microbiome-based therapeutics. From an engineering perspective, this heterogeneity imposes stringent requirements on predictive models, including robustness to inter-individual variability, capacity for longitudinal simulation, and the ability to generalize across intervention types.

Compared with purely predictive classifiers, AI-driven simulation models—often referred to as “digital twins”—aim to replicate an individual’s microbiome and its interactions with host physiology within a computational framework [34]. These models typically integrate multi-omic data—such as metagenomics, metabolomics, and host transcriptomics—and employ machine learning methods to forecast how interventions might reshape microbial composition, metabolic outputs, and downstream host phenotypes [35]. For example, frameworks such as Q-net and MICOM emerged in biological studies. Q-net is a temporal machine learning framework that constructs digital twins of the infant microbiome by modeling longitudinal microbial trajectories, enabling the prediction of growth and neurodevelopmental outcomes under probiotic interventions. The Q-net platform developed at the University of Chicago constructs digital twins of the infant microbiome and predicts their response trajectories, such as how probiotic supplementation might mitigate neurodevelopmental risk, achieving 76% accuracy in forecasting growth outcomes [36]. Similarly, MICOM is a community-scale metabolic modeling platform that integrates genome-scale metabolic models with microbiome abundance data to simulate metabolic fluxes and predict individualized metabolite production, such as short-chain fatty acids. It enables ensemble learning simulations of probiotic or dietary interventions, revealing personalized shifts in short-chain fatty acid production after antibiotic disruption [37]. By leveraging these in silico simulations, researchers can pre-screen candidate interventions, reduce trial-and-error in lab and clinical settings, and prioritize promising strategies before committing to resource-intensive in vivo trials. These approaches prioritize mechanistic interpretability and intervention simulation, but require dense longitudinal and multi-omics data, which can limit scalability in routine clinical settings.

Several pioneering studies have demonstrated the utility of AI in predicting individual responses to microbiome-targeted therapies. In inflammatory bowel disease (IBD), for instance, machine learning models trained on patient-specific microbiome and clinical profiles have been used to stratify patients into likely responders and non-responders to FMT or probiotic regimens [38]. These models often incorporate longitudinal data to capture temporal fluctuations in microbial dynamics and identify predictive microbial biomarkers associated with treatment efficacy [39]. Similarly, deep learning approaches are being explored to simulate community-level dynamics under interventions such as dietary changes, drug exposure, or microbial supplementation, providing insights into ecosystem stability, resilience, and optimal intervention windows [40].

Beyond predicting therapeutic response, AI can be leveraged to design novel microbiome-targeted strategies by navigating the vast combinatorial spaces of microbial strain blends, diet–microbe interactions, and prebiotic formulations. For instance, generative models and evolutionary algorithms have been applied to optimize microbial consortia capable of enhancing short-chain fatty acid (SCFA) production, mitigating inflammation, or promoting colonization resistance [41,42]. Many of these platforms integrate genome-scale metabolic models (GEMs)—which encapsulate microbial metabolic networks—into AI-assisted workflows, such as constraint-based reconstruction and analysis (COBRA), to simulate how microbial interactions affect metabolite fluxes in host–microbiome systems [43]. These hybrid modeling frameworks facilitate hypothesis generation for next-generation microbial therapeutics, empowering researchers to design interventions driven by functional outcomes rather than taxonomic profiles. For example, tools like the PROSO Toolbox utilize protein-constrained GEMs in silico to identify gene and pathway targets for strain optimization, while platforms like COMETS enable spatiotemporal simulations of microbial ecosystems under diverse environmental interventions [44].

Ultimately, AI-powered microbiome simulation represents a paradigm shift from empirical treatment selection toward data-driven, personalized therapy design. These in silico models allow virtual testing and optimization of microbiome-modulating interventions, improving the precision, safety, and efficacy of therapeutic strategies before real-world implementation [45,46]. They also offer an ethically sound, cost-effective bridge between laboratory discovery and clinical translation—reducing reliance on animal models and minimizing risk by predicting potential adverse outcomes in silico. As the availability of high-resolution longitudinal microbiome datasets increases and simulation frameworks begin integrating host factors like immune profiles, pharmacokinetics, and lifestyle data, AI-based therapeutic optimization is poised to become a cornerstone of personalized microbiome medicine, aligning with broader advances in precision health [14,33].

Table 1 highlights key studies (2015–2025) demonstrating how artificial intelligence has advanced microbiome-based therapeutic optimization across diet, probiotics, FMT, and pharmacotherapy. Early work by Zeevi et al. (2015) [47] and Deehan et al. (2020) [48] applied machine learning to personalize nutrition and optimize fiber-derived metabolites. Subsequent studies, including Westfall et al. (2021) [49] and McCoubrey et al. (2021) [50], used adaptive regression and ensemble modeling to design probiotic formulations and predict drug–microbiome interactions. Recent frameworks, such as Shtossel et al. (2023) [51] and Quinn-Bohmann et al. (2024) [52], integrated deep learning, genetic algorithms, and metabolic modeling to forecast FMT outcomes and individual SCFA production. Large-scale studies by Murovec et al. (2024) [53], Pateriya et al. (2025) [54], and Nie et al. (2025) [55] further applied AutoML and time-series prediction for disease screening and global CRC forecasting. Together, these studies illustrate AI’s evolution from predictive modeling to mechanistic and multi-omics frameworks enabling precision microbiome therapeutics.

Viewed comparatively, the studies summarized in Table 1 reveal a clear hierarchy of modeling strategies and design trade-offs relevant to therapeutic optimization. Early machine-learning approaches demonstrate scalability and strong predictive performance across cohorts but are primarily correlational, limiting their utility for intervention design. In contrast, digital twin and community-scale metabolic modeling frameworks (e.g., Q-net, MICOM, MCMM) enable mechanistic simulation of microbiome responses to dietary, probiotic, or pharmacological perturbations, supporting in silico testing and optimization of interventions. However, these approaches require longitudinal data, curated metabolic reconstructions, and increased computational complexity, which currently constrain clinical scalability. Hybrid strategies that integrate mechanistic constraints with data-driven learning appear most promising, as they balance predictive accuracy, biological interpretability, and translational feasibility. From a bioengineering standpoint, these trends suggest a shift from single-cohort prediction toward simulation-enabled intervention design as models mature.

Collectively, these findings indicate that therapeutic microbiome AI is transitioning from descriptive prediction toward simulation-based control and optimization, aligning the field more closely with core bioengineering principles of system modeling, intervention design, and performance trade-off analysis.

## 3. Personalized Nutrition and Precision Medicine

How can AI models be designed to capture individualized diet–microbiome–host interactions while balancing predictive accuracy, interpretability, scalability, and clinical deployability? The interplay between diet and the gut microbiome exemplifies a profound form of gene–environment interaction in human biology, wherein dietary inputs shape microbial ecosystems that, in turn, exert extensive downstream effects on host metabolism, immune regulation, and neurological function [59,60]. For example, metabolites produced from dietary fiber by gut microbes—including short-chain fatty acids—have been shown to modulate glycemic control, inflammatory responses, and even brain and eye health via the gut–brain axis [5,61,62,63]. However, individuals differ substantially in both their baseline microbiome compositions and their microbial responses to identical diets, posing a significant barrier to one-size-fits-all nutrition strategies. In this context, artificial intelligence—especially machine learning and deep learning—offers powerful tools for deciphering these complex, personalized diet–microbiome interactions and for developing precision nutrition approaches tailored to the individual [15,40,64]. This problem requires models that can integrate heterogeneous inputs, generalize across individuals, and generate actionable recommendations under real-world clinical constraints.

AI-driven precision nutrition frameworks combine high-throughput microbiome profiling with dietary intake records, clinical biomarkers, and lifestyle data to model and predict individualized physiological responses to specific foods. This approach was first popularized by the landmark study of Zeevi et al. (2015), which demonstrated that ensemble learning models integrating microbiome and clinical data can achieve strong predictive accuracy at scale [47]. However, such models rely primarily on correlational associations and require careful validation to ensure generalizability beyond the original cohort. Their randomized controlled trial demonstrated that personalized dietary recommendations based on these predictions significantly improved glycemic control compared to standardized diets [47]. Since then, similar models have expanded to other dietary interventions, including macronutrient compositions such as fiber types and fermented foods. For example, Deehan et al. (2020) identified how discrete dietary fiber structures shape SCFA production using AI-guided metabolic modeling across microbiome profiles [48]. Wastyk et al. (2021) reported that fermented food intake modulates immune status via microbiome changes, an effect quantified by deep learning–augmented statistical analysis [56]. These frameworks seek to optimize gut microbial diversity, enhance beneficial metabolite production, and reduce inflammation through tailored dietary interventions [56]. In a recent randomized trial, Kouraki et al. (2024) applied machine learning to profile both stool and serum metabolomics following six weeks of inulin or omega-3 supplementation in 64 healthy adults [57]. Using elastic net regression and random forest models, they identified specific metabolites—indole propionate for fiber and CMPF and EPA for omega-3—that distinguished individual responses, partially explained by gut microbiome composition shifts such as increased abundance of *Coprococcus* [57]. Together, these predictive models enable the formulation of personalized diet plans that are both effective and sustainable, by accounting for the unique microbiome–metabolism interface of each individual. Furthermore, comprehensive models that integrate microbiome, nutritional, and lifestyle data outperform standard dietary recommendations, moving precision nutrition toward real-world implementation. The studies summarized in Table 2 highlight distinct modeling strategies for personalized nutrition, each occupying a different region of the design space. Ensemble machine-learning models trained on large cohorts offer scalability and robust performance for population-level personalization but provide limited mechanistic insight into diet–microbiome interactions. In contrast, metabolic and community-scale modeling approaches enable functional prediction of microbial metabolite production and diet-specific responses, supporting mechanistic interpretation and intervention design, but require richer multi-omics data and careful curation. Longitudinal and multi-modal frameworks further enhance personalization by capturing temporal dynamics and host context, yet introduce challenges related to data availability, interpretability, and clinical integration. From a bioengineering standpoint, these trade-offs suggest that hybrid architectures—combining scalable learning with biologically informed constraints—represent the most promising pathway toward clinically deployable precision nutrition systems.

In addition to dietary guidance, AI models are also being employed to predict therapeutic outcomes in the broader context of precision medicine [17,68]. Gut microbiota has been shown to modulate the efficacy and toxicity of numerous drugs, including chemotherapeutics, immunotherapies, and psychiatric medications [7,33,56]. By integrating microbiome data with pharmacokinetic parameters and host genotypes, AI can help forecast individualized drug responses, identify likely adverse effects, and suggest microbiome-modulating co-therapies to improve outcomes [14,68]. For example, in oncology, studies have linked the composition of gut microbes to patient response rates to immune checkpoint inhibitors; AI models are being developed to stratify patients and guide probiotic or dietary preconditioning before treatment [17,19,69]. This approach exemplifies the promise of microbiome-informed pharmacotherapy, where microbial profiles guide not just what patients eat, but also how they are treated.

Another key advantage of AI in personalized nutrition and precision medicine lies in its ability to integrate multi-modal data [70,71]. Beyond taxonomic data from 16S or metagenomic sequencing, researchers are incorporating microbial transcriptomics, metabolomics, host genotyping, continuous glucose monitoring, and wearable device data into unified predictive frameworks [72,73]. These multi-omic and digital phenotyping datasets are often too high-dimensional and noisy for conventional analysis, but AI models—especially ensemble learning and multi-modal deep learning architectures—are well-suited to extracting biologically meaningful patterns [72]. This allows for the generation of actionable insights that are both individualized and grounded in mechanistic understanding, moving beyond correlation-based personalization toward causally informed, functionally optimized intervention strategies.

Together, these developments mark a significant transition toward microbiome-informed healthcare, in which AI serves as the computational bridge between complex biological data and real-world clinical decisions [69,72]. By tailoring nutrition and therapeutic interventions to the individual’s microbiome profile, AI enables a more effective, preventive, and participatory model of medicine. While challenges remain—such as the need for larger, more diverse training datasets, improved interpretability of black-box models, and integration into clinical workflows—the trajectory is clear. AI-driven personalization of nutrition and treatment has the potential to transform healthcare from population averages to microbiome-aware precision care, fundamentally redefining our approach to diet, disease, and wellness.

**Figure 2:**
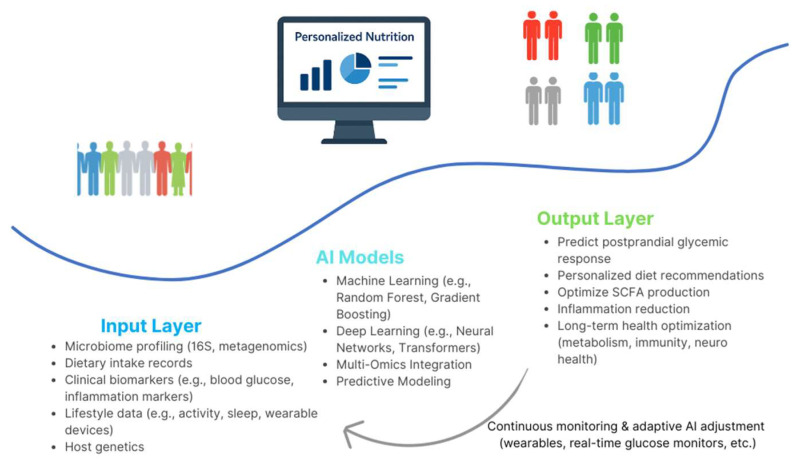
AI-Driven Personalized Nutrition from Microbiome Data.

Figure 2 operationalizes an AI-driven personalized nutrition pipeline, illustrating how heterogeneous inputs are integrated, modeled, and translated into actionable dietary and therapeutic recommendations under a closed-loop feedback framework. The process begins with comprehensive multi-modal input data, including microbiome profiling (via 16S rRNA or metagenomics), dietary intake records, clinical biomarkers (such as glucose and inflammatory markers), lifestyle factors (activity, sleep, wearables), and host genetic information. These data streams are integrated through sophisticated machine learning and deep learning models—including ensemble algorithms, neural networks, and multi-omics frameworks—that can predict individualized metabolic responses and therapeutic needs. The outputs of such models generate actionable recommendations: predicting postprandial glycemic responses, formulating personalized dietary interventions, optimizing short-chain fatty acid production, reducing inflammation, and promoting long-term metabolic, immune, and neurocognitive health outcomes. The adaptive nature of these frameworks allows continuous monitoring and real-time refinement via feedback loops incorporating wearable technologies and continuous glucose monitoring devices. Taken together, this evolving paradigm demonstrates how AI-enabled multi-omics integration can bridge complex diet–microbiome–host interactions, translating biological complexity into individualized, actionable nutrition and treatment plans. As such, AI-driven personalized nutrition represents a key frontier in the future of precision medicine.

A hierarchy of methodological maturity is evident across personalized nutrition studies, with single-cohort predictive models representing early feasibility, and externally validated, longitudinal, and multi-omics-integrated frameworks constituting higher levels of translational readiness. Overall, AI-driven personalized nutrition evolves from cohort-specific prediction toward systems-level modeling and adaptive intervention design, reinforcing its alignment with core bioengineering principles of system integration, optimization, and control.

## 4. Public Gut Microbiome Datasets for AI Research

What data characteristics are required to train, validate, and benchmark generalizable and clinically reliable AI models for gut microbiome applications? There is a lot to consider, such as scale, diversity, longitudinal depth, and annotation richness. More and more datasets are available for gut microbiome research in recent years. These datasets establish baseline reference distributions and enable model calibration, but their limited population diversity and intervention coverage constrain generalizability for clinical deployment. A foundational resource supporting AI-driven gut microbiome research is the HMP, a landmark initiative that generated over 2000 metagenomic samples (primarily 16S and whole-genome shotgun sequences) from a healthy adult cohort, accompanied by extensive host metadata including dietary records, immune status, and metabolic profiling [25]. HMP not only provides baseline microbial community data across body sites but also enables AI algorithms to learn from large, well-curated reference sets. Its second phase, the Integrative HMP, enriched the resource with longitudinal datasets spanning respiratory, vaginal, IBD, and type 2 diabetes, offering temporal and disease-specific data valuable for modeling microbial dynamics and predictive AI workflows [74].

Complementing HMP, the NIH Common Fund’s Healthy Human Reference Dataset aggregates over 10 terabytes of metagenomic data, representing one of the most comprehensive collections of shotgun-sequenced healthy microbiomes [75]. This extensive dataset supports the development and validation of AI models requiring large sample sizes and heterogeneity across populations. Similarly, publicly curated platforms like MG-RAST and MGnify offer thousands of annotated gut microbiome samples from diverse studies, with open APIs and analysis pipelines, facilitating standardized processing, cross-study benchmarking, and federated AI model development [76,77].

Collectively, public gut microbiome datasets serve distinct engineering roles: large consortia datasets primarily support model training and representation learning; curated multi-omics cohorts enable mechanistic inference and intervention modeling; and harmonized repositories facilitate cross-study benchmarking and external validation. Distinguishing these roles is essential for appropriate model development and evaluation.

Recent initiatives have focused on assembling multi-omics paired datasets—an essential input for advanced AI models integrating microbiome, metabolome, and host data [78,79,80,81]. A notable example is the curated gut microbiome–metabolome resource, which compiles over 10 comprehensive human fecal studies including genomic, metabolic, and phenotypic information, all normalized and formatted for AI-ready analysis [82]. These integrated repositories enable more sophisticated machine learning applications, such as joint modeling of microbial taxa and metabolic outputs, for tasks like predicting short-chain fatty acid production based on gut composition and dietary context [48].

Beyond large-scale consortia, specialized datasets add depth and breadth to training corpora for AI tasks. The Gut Virus Catalog includes 189,680 viral genomes derived from over 11,000 stool metagenomes and captures hundreds of thousands of viral proteins, offering valuable insight into the virome—a less-explored component of gut ecology [83]. Other domain-specific collections include the Mustard Database, which tracks over 6000 antimicrobial-resistance determinants in gut microbes—a resource crucial for AI models analyzing resistome features and their implications for personalized therapeutics [84].

Moreover, open repositories such as Kaggle’s Human Metagenomics dataset (3000+ metagenomes) and community resources like GMrepo, which hosts consistently curated human gut metagenomes, enhance accessibility and comparability across cohorts [85,86,87]. These platforms are designed to ease data retrieval, filtering, and normalization—key steps for AI-driven modeling. By leveraging such rich, standardized datasets spanning healthy and disease contexts, researchers can train, validate, and compare AI models at scale, ultimately accelerating progress in microbiome-based diagnostic, therapeutic, and nutritional applications. Table 3 provides a comparative overview of key publicly available gut microbiome datasets that support AI research and development. These datasets vary widely in sample size, data types (e.g., 16S rRNA sequencing, whole-genome shotgun metagenomics, metabolomics), and metadata richness, enabling a range of machine learning tasks from supervised classification to unsupervised clustering and longitudinal modeling. Flagship resources such as the HMP [25] and MGnify serve as foundational references, while more specialized collections like the Gut Virome Database (GVD) and the Mustard Database offer niche insights into less-studied components like viral populations and antimicrobial resistance. By leveraging these datasets, researchers can build scalable, generalizable, and reproducible AI models tailored to diagnostic, therapeutic, or ecological applications in gut microbiome science.

As summarized in Table 3, public gut microbiome datasets vary substantially in scale, annotation depth, and longitudinal structure, leading to important trade-offs for AI model development. Large-scale amplicon and shotgun metagenomic collections enable robust representation learning and transfer learning but often lack detailed phenotypic or intervention metadata. In contrast, smaller longitudinal and multi-omics cohorts provide richer biological context and support causal or mechanistic modeling, yet are more susceptible to overfitting and cohort-specific bias. From a bioengineering standpoint, model benchmarking should therefore be interpreted relative to dataset characteristics, with no single dataset serving as a universal gold standard. Effective evaluation increasingly requires multi-dataset validation spanning reference, disease-specific, and longitudinal cohorts.

Metabolomics datasets provide a critical functional bridge between microbial composition and host physiology, enabling AI models to move beyond taxonomic associations toward mechanistic and predictive interpretation. Public resources such as the IBD Multi’omics Database (IBDMDB; iHMP/HMP2) offer longitudinal fecal metabolomics integrated with metagenomics, metatranscriptomics, proteomics, and clinical metadata, allowing AI frameworks to model disease dynamics and treatment responses over time. Complementing this, the gut microbiome–metabolome dataset collection aggregates paired microbiome and metabolomics data across multiple cohorts, facilitating AI-driven discovery of reproducible microbe–metabolite relationships and benchmarking of integration methods. Broader metabolomics repositories, including MetaboLights and the Metabolomics Workbench/MetabolomeXchange ecosystem, further expand access to gut-related metabolomics studies and support large-scale training and validation of machine learning models. Together, these datasets enable AI to transform metabolomics from descriptive metabolite profiling into predictive and functional modeling of host–microbiome interactions, addressing a key limitation of traditional microbiome analyses.

A hierarchy of data readiness emerges across public microbiome resources. Cross-sectional taxonomic datasets support early-stage model development and feature discovery, whereas longitudinal and multi-omics datasets represent higher tiers of engineering readiness by enabling temporal prediction, intervention modeling, and validation of mechanistic hypotheses. Clinical translation of microbiome AI systems will increasingly depend on datasets that combine longitudinal sampling, standardized metadata, and diverse population representation.

## 5. Cutting-Edge Techniques for AI in Gut Microbiome Research

Which AI architectures provide the most favorable trade-offs among predictive accuracy, interpretability, data efficiency, and translational readiness for gut microbiome applications? The following subsections examine complementary AI architectures through the lens of system-level design considerations, highlighting where each approach excels, where it fails, and how these methods can be combined in practical microbiome AI pipelines.

### 5.1. Transformer-Based Microbiome Language Models

Inspired by the success of transformer architectures in natural language processing, recent studies treat gut microbiome profiles as a “language” to be understood and modeled [21,88]. Quintin Pope et al. (2025) trained a self-supervised transformer model on large, unlabeled gut microbiome datasets, such as the American Gut data, to learn contextualized embeddings for microbial taxa and entire samples [89]. These embeddings capture taxon–taxon co-occurrence and ecological patterns analogous to word contexts in sentences. Transformer-derived representations, when used in downstream tasks like IBD detection and dietary pattern classification, outperform traditional approaches and exhibit superior generalization to unseen cohorts [88]. Biological interpretability is further enhanced, as embedding spaces reflect phylogenetic relationships and known metabolic pathways without supervision. Thus, transformer-based models offer a powerful and flexible tool for generating biologically meaningful representations from vast microbial datasets.

Beyond static representations, recent work has begun to explore temporal transformer models that incorporate longitudinal microbiome data to capture dynamic ecosystem shifts over time [21,90]. Such approaches are particularly valuable in tracking disease progression, treatment response, and microbial resilience following interventions. Furthermore, attention-based transformers have been applied to integrate multiple hierarchical layers of microbiome information—including species abundance, gene function, and metabolic pathways—yielding multi-scale representations that enhance both prediction accuracy and biological interpretability [91,92]. For example, MetaTransformer leverages self-attention mechanisms to directly model sequencing reads, enabling taxonomic and functional annotation from raw metagenomic data without extensive preprocessing [92]. This capacity to jointly model sequence-level and community-level information positions transformer-based models as a uniquely versatile framework for gut microbiome research, with the potential to unify taxonomic profiling, functional annotation, and host-microbe interaction prediction within a single scalable architecture.

Transformer-based microbiome language models are particularly well-suited for large-scale representation learning and transfer learning across cohorts, as they efficiently leverage unlabeled data to learn ecological context. However, their reliance on large training corpora and abstract embedding spaces introduces challenges for interpretability and clinical validation. Consequently, these models are most effective as upstream feature extractors rather than standalone decision systems, serving as foundational layers within larger, task-specific pipelines.

### 5.2. Sequence-Level Language Models for Microbial Genomes

Transformer architectures are now being leveraged to model raw microbial genetic content [91,93,94]. Protein and gene sequences in microbiomes can be regarded as the literal “language of life,” and natural language-inspired approaches are proving valuable. Methods such as MetaLLM and other large-scale sequence-based transformers learn representations from millions of microbial protein or gene sequences, enabling tasks such as functional annotation, enzyme discovery, and virome analysis [95]. These models not only improve classification accuracy across taxonomic and functional tasks but also support cross-species transfer learning, facilitating broader and deeper microbiome analyses [88,93].

New techniques have also demonstrated that pre-trained large language models, originally developed for natural language, can be fine-tuned on microbial sequence data to extract functional and evolutionary insights at an unprecedented scale. For instance, Shishir et al. (2023) developed MetaLLM, a deep transformer model capable of predicting metal ion binding residues directly from protein sequences, illustrating how sequence-based transformers can capture intricate biochemical properties relevant to microbial metabolism and host interactions [95]. This capacity to predict functional sites, enzyme activity, or antimicrobial resistance from raw sequences offers new avenues for exploring the mechanistic roles of gut microbiota in health and disease. As increasingly large protein and gene databases become available, sequence-level transformer models are expected to further enhance our understanding of functional diversity across the gut microbiome and accelerate biomarker discovery for clinical translation [92,95].

Sequence-level transformer models address a distinct engineering layer by operating directly on microbial genetic content rather than community composition. These models excel in functional annotation and enzyme discovery, but are less directly applicable to host-level phenotype prediction without aggregation or integration layers. In practice, they are most valuable when coupled to higher-level community or host models, enabling multi-scale pipelines that link molecular function to clinical outcomes.

### 5.3. Graph Neural Networks on Phylogenetic and Metabolic Structures

Graph neural networks (GNNs) natively capture the non-Euclidean and relational nature of microbiome data and also play key roles in new ways to explore biological meanings. Irwin et al. (2024) introduced a GNN that models microbial taxa as nodes connected by phylogenetic or co-occurrence edges, generating embeddings tuned to disease prediction tasks [96]. By incorporating network topology and taxon relationships, GNNs can better capture community structure than flat ML models. This framework is especially effective for predicting host phenotypes like IBD, showcasing another way AI can leverage biological structure to enhance performance [96].

Beyond taxonomic networks, functional and metabolic graphs are increasingly being integrated into GNN frameworks to model gut microbial ecosystems at a systems level. Rehman et al. (2024) proposed a GNN approach that incorporates drug–microbiome interaction networks, where nodes represent microbial species and edges encode shared metabolic pathways or drug susceptibility profiles [58]. This enables the prediction of how microbiome composition may influence pharmacological outcomes, advancing precision pharmacotherapy. Similarly, multi-layer GNNs that simultaneously model phylogenetic, metabolic, and ecological relationships offer a powerful means to uncover higher-order community interactions that drive host phenotypes and treatment responses [97]. As more comprehensive multi-omic datasets become available, GNN-based models are poised to play a central role in linking microbial network structure with clinical applications in microbiome-informed medicine.

Compared with transformer-based models, GNNs explicitly encode biological structure into the learning process, improving interpretability and alignment with biological knowledge. However, GNN performance depends critically on graph construction quality and prior knowledge availability, which can limit scalability across datasets. From a design standpoint, GNNs are most effective in scenarios where relational structure is well defined, and mechanistic insight is prioritized over raw predictive performance.

### 5.4. Multi-Modal Learning for Microbiome and Host Integration

A burgeoning area of development is multi-modal AI, where microbiome data is combined with complementary datasets such as histological images, dietary logs, metabolomics, genomics, and clinical measurements [14,69,98]. One notable study integrated fecal microscopy images with shotgun metagenomic profiles using convolutional neural networks and other AI techniques, achieving high accuracy in predicting the abundance of short-chain fatty acid–producing taxa [99]. These models also provided interpretable visual cues by highlighting microscope image regions correlated with predictions [14]. Other teams have fused omics and clinical metadata within deep, multi-modal architectures—such as integrative transformer or attention-based models—demonstrating superior predictive power for complex traits like metabolic syndrome compared to models using only one data modality [71,78,80]. Multi-modal integration enables more comprehensive and clinically relevant models by encompassing interactions across biological layers.

More recently, multi-modal fusion models incorporating longitudinal and real-time data streams have emerged to further enhance predictive accuracy and clinical utility. For example, Tangaro et al. (2024) developed an explainable AI framework that integrates microbiome, metabolome, and continuous wearable sensor data to dynamically predict disease states such as Behçet’s disease [98]. Such platforms allow for time-sensitive monitoring of host–microbiome interactions, enabling adaptive interventions and personalized disease management. Moreover, transformer-based multi-modal models, such as OmicsFormer, have demonstrated superior ability to integrate diverse data types—ranging from host transcriptomics to dietary logs—yielding systems-level insights that improve both phenotype prediction and mechanistic interpretation [100]. As the volume and diversity of health-related data increase, multi-modal AI approaches are likely to play a pivotal role in real-world precision medicine applications, facilitating continuous monitoring, early detection, and proactive microbiome-targeted interventions.

Multi-modal AI frameworks represent a critical step toward clinical deployment by integrating microbiome data with host phenotypes, imaging, and behavioral signals. While these models consistently outperform single-modality approaches, they introduce practical challenges related to data synchronization, missing modalities, and increased system complexity. Engineering robust multi-modal systems therefore requires modular architectures, flexible data fusion strategies, and careful evaluation of modality-specific contributions.

### 5.5. Integrated Modular Frameworks for Multi-Omics Analysis

As we know, handling multiple omics types (e.g., metagenomics, transcriptomics, metabolomics, and host clinical features) poses significant computational challenges [101,102]. To address this, Muller et al. (2024) developed MintTea, an integrative pipeline using canonical correlation analysis and consensus clustering to identify disease-associated multi-omic modules [103]. Applied to metabolic syndrome and colorectal cancer cohorts, MintTea identified modules containing co-varying microbial species and metabolites—such as Peptostreptococcus with elevated amino acids in colorectal cancer—that provide systems-level insights beyond single-omic associations [103]. Furthermore, transformer-based multi-omics architectures—e.g., “OmicsFormer”—combine numerical and sequence features into unified models, achieving improved disease prediction accuracy across multiple data types [100]. These integrated frameworks enable hypothesis generation and reveal mechanistic relationships across diverse omics layers.

**Figure 3:**
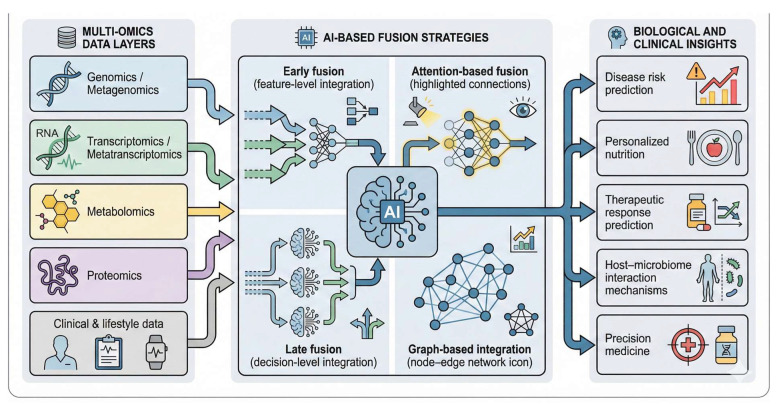
AI-enabled multi-omics integration frameworks in gut microbiome research.

Furthermore, building on these frameworks, network-based multi-omics integration has gained attention for its ability to model complex interdependencies between microbial taxa, metabolites, and host factors. Wu and Xie (2025) introduced an AI-driven multi-scale network integration approach that links genotype, environment, and phenotype relationships using layered multi-omics data [78]. By constructing heterogeneous graphs where nodes represent diverse biological entities (e.g., microbial species, metabolites, host genes), and edges capture their functional or causal relationships, such models can infer cross-domain interactions driving disease phenotypes. Similarly, Biswas and Chakrabarti (2020) demonstrated how AI-based systems biology approaches can integrate multi-omics data to reveal novel biomarkers and mechanistic insights in cancer, showcasing the broader applicability of these methods beyond gut microbiome research [79]. These emerging integrative frameworks exemplify how AI can leverage biological network structures across omics layers to enhance both discovery and translational potential in microbiome-informed medicine. Figure 3 illustrates representative AI-enabled multi-omics integration architectures, highlighting how diverse omics layers are fused using complementary AI strategies to generate biological and clinical insights. For example, metagenomic data may suggest the presence of short-chain fatty acid–producing microbes, while AI-integrated metatranscriptomic and metabolomic analyses reveal that these pathways are functionally inactive. By jointly modeling multi-omics and clinical data, AI uncovers patient-specific metabolic disruptions that explain variable dietary responses and enable targeted nutritional or therapeutic interventions.

### 5.6. Generative and Diffusion-Based Models for Data Imputation

Advanced generative AI models, such as diffusion models and variational autoencoders (VAEs), are now being applied to microbiome datasets [21,104]. Shi et al. (2024) introduced mbVDiT, a conditional diffusion model that imputes missing microbiome data and denoises noisy samples, guided by patient metadata and leveraging pre-trained latent distributions [90]. This method significantly outperformed classic imputation strategies in cancer-related microbiome datasets [90]. Generative models hold promise for data augmentation, synthetic sample generation, and domain adaptation in microbiome AI, especially when labeled data are sparse or highly imbalanced.

To complement the discussion of emerging AI technologies in gut microbiome research, Table 4 provides an updated overview of both current and next-generation methodologies driving innovation in this field. The table summarizes a broad spectrum of approaches—from transformer language and sequence-based models that capture microbial context and function, to graph neural networks that encode phylogenetic and metabolic relationships. It also highlights multi-modal and multi-omics frameworks that integrate microbiome, host, and environmental data, as well as generative models that address data sparsity through diffusion and variational autoencoder architectures. Recent methodological advances such as causal and explainable AI, federated learning, digital twin modeling, and AutoML pipelines further expand the analytical frontier, emphasizing interpretability, data security, and scalability. Collectively, these techniques represent a rapidly evolving methodological ecosystem that enhances predictive accuracy, mechanistic insight, and translational relevance in AI-driven gut microbiome research.

### 5.7. Architectural Trade-Offs and System Design Considerations

Across the techniques reviewed above, no single AI architecture dominates across all microbiome applications. Transformer models offer scalability and representation power but require large datasets and careful interpretability strategies. Graph neural networks provide structure-aware learning at the cost of graph dependency and reduced flexibility. Multi-modal systems enhance clinical relevance while increasing engineering complexity, and generative models improve robustness under data sparsity but introduce synthetic uncertainty. From a bioengineering standpoint, effective microbiome AI systems increasingly adopt hybrid architectures that combine representation learning, mechanistic constraints, and modular integration to balance accuracy, interpretability, and translational readiness.

## 6. Current Challenges, Limitations, and a Prioritized Research Agenda

Despite rapid advances in applying AI to gut microbiome research, the field faces persistent challenges rooted in the unique characteristics of microbiome data. From a bioengineering perspective, these challenges are not equally limiting and must be addressed in a prioritized manner to enable reproducible and clinically translatable systems.

### 6.1. Priority 1: Data Heterogeneity, Sparsity, and Standardization

The most fundamental bottleneck in microbiome AI remains data heterogeneity and sparsity. Most notably, gut microbial datasets are high-dimensional and sparse: a single sample can contain thousands of taxa or gene features, many of which are rare or absent in other individuals [109]. This results in a “curse of dimensionality” where the number of features far exceeds the number of samples, leading to overfitting in many machine learning models [18]. The situation is exacerbated by the relatively small cohort sizes of most microbiome studies, especially those involving multi-omics or longitudinal designs. This imbalance complicates model training and validation, reducing generalizability and increasing the risk of reporting inflated performance metrics that fail to hold in independent cohorts.

In addition to data complexity, confounding factors and inter-individual variability pose serious obstacles to AI-driven discovery. Gut microbiome composition is highly sensitive to external influences such as diet, medication use, geography, age, lifestyle, and host genetics [18,107]. Without rigorous control or adjustment for these variables, AI models may inadvertently learn patterns associated with confounders rather than true disease signals [109]. As a result, models may fail when transferred across cohorts or deployment contexts.

Another critical limitation is the lack of consensus on best practices for microbiome data preprocessing, feature engineering, and model validation [109,110]. Differences in sequence processing pipelines can lead to substantial variations in the resulting feature matrices, even when analyzing identical raw data. The absence of standardized benchmarking frameworks further hinders reproducibility and cross-study comparison, representing a first-order engineering constraint.

### 6.2. Priority 2: Limited Interpretability and Mechanistic Insight

Beyond data limitations, model interpretability represents a major barrier to trust and translation. Many AI models are criticized for being opaque or “black-box” systems, offering limited biological interpretability [105,106]. While they may achieve high accuracy in phenotype prediction, these models often provide little insight into which microbial taxa, pathways, or interactions are driving the prediction. This is a serious drawback in a biological and clinical context, where understanding mechanistic relationships is as important as performance metrics.

Although tools like SHAP values, feature importance scores, and attention maps are increasingly used to extract interpretable signals from complex models, these approaches are still under development and require careful validation to ensure biological plausibility [111]. From an engineering standpoint, interpretability must be treated as a design requirement rather than a post hoc add-on.

### 6.3. Priority 3: Data Sparsity and Longitudinal Modeling

The predominance of cross-sectional datasets limits the ability of AI systems to model temporal dynamics and intervention effects. Most microbiome datasets provide static snapshots, constraining causal inference and prediction of treatment response [112,113]. This limitation reduces the utility of AI for therapeutic optimization and personalized intervention design.

Future progress will depend on expanded longitudinal cohort designs and time-aware modeling frameworks. Simulation-based approaches, including digital twin models, offer a promising pathway to leverage sparse temporal data while enabling in silico testing of interventions [10,33,34].

### 6.4. Priority 4: Population Bias, Equity, and Generalizability

Broader concerns regarding bias and data equity further limit the applicability of current microbiome AI systems [111,114]. Many publicly available microbiome datasets disproportionately represent individuals from North America and Europe, raising concerns about the generalizability of AI models trained on these populations to other ethnic or geographic groups [115,116]. Models trained on narrow population distributions risk systematic failure when applied globally. Furthermore, most datasets represent cross-sectional snapshots, limiting the capacity of AI systems to infer causal relationships or temporal dynamics [115]. Regulatory, ethical, and privacy issues related to microbiome data usage—especially when combined with host genomic and clinical information—also remain underexplored [108,117]. These limitations must be addressed through larger, more diverse and standardized datasets; development of interpretable and generalizable AI methods; and closer collaboration between computational scientists, microbiologists, and clinicians. Only then can AI’s full potential in gut microbiome research be realized in clinical practice.

### 6.5. Priority 5: Clinical Translation, Privacy, and Deployment Barriers

Even well-performing models face significant barriers to real-world deployment. Regulatory, ethical, and privacy concerns related to microbiome and host data usage remain underexplored [108]. Without privacy-preserving infrastructures and workflow integration, many AI tools will remain confined to research settings. Federated learning, decentralized benchmarking, and shared open-source infrastructures offer promising solutions for enabling global collaboration while preserving patient confidentiality.

### 6.6. Outlook: Toward an Engineering-Driven Roadmap

As artificial intelligence continues to evolve, its integration with gut microbiome research is expected to become increasingly sophisticated and clinically relevant [14,16,64]. Near-term priorities include real-time and adaptive AI systems capable of monitoring microbiome dynamics, while mid-term advances will depend on simulation-enabled intervention design and longitudinal modeling [14].

Another key frontier is the integration of AI-driven microbiome modeling into clinical trial design and therapeutic pipelines [106]. Rather than treating the microbiome as a passive biomarker, future research may use AI to simulate and predict microbial responses to candidate drugs, optimize probiotic or dietary formulations, or stratify patients for personalized therapeutic regimens [99,110,118]. In silico simulations could substantially reduce the cost and time required for microbiome-targeted drug development. Furthermore, advances in causal inference and mechanistic AI could enable researchers to move beyond correlation-based insights toward identifying true microbial drivers of disease and health. In addition to computational modeling of microbiome dynamics, AI is increasingly applied to related fields such as material design and medical biosensors. For example, recent advances in colloidal nanoribbons and nanostructures have enabled the development of highly sensitive optoelectronic devices [119,120,121]. These technologies may provide next-generation platforms for real-time microbiome monitoring through nano-enabled biosensing, offering opportunities for tighter integration between AI-driven modeling and experimental sensing capabilities.

Moreover, progress in microbiome AI will require a shift from descriptive modeling toward systems-level engineering, guided by prioritized constraints, measurable technical milestones, and translational readiness. As these technological, ethical, and infrastructural components mature in parallel, AI-guided microbiome research is poised to evolve from a niche innovation into a central pillar of 21st-century precision medicine.

## 7. Conclusions

The integration of artificial intelligence into gut microbiome research marks a pivotal shift in how we analyze, interpret, and ultimately apply microbial data to human health. From early machine learning classifiers to advanced transformer models and multi-modal deep learning frameworks, AI has evolved into an indispensable tool capable of uncovering subtle patterns across complex, high-dimensional, and heterogeneous microbiome datasets. These tools have enabled the discovery of disease-associated microbial signatures, the prediction of individual therapeutic responses, and the personalization of dietary and pharmacological interventions. By simulating microbial dynamics, integrating multi-omics data, and capturing host–microbiome interactions, AI is transforming our capacity to generate clinically actionable insights from microbiome profiles.

At the forefront of this transformation are emerging techniques such as microbiome language models, graph neural networks, generative architectures, and multi-modal fusion methods, all of which push beyond traditional taxonomic analysis toward functional and systems-level understanding. These advances not only improve prediction accuracy but also offer opportunities for mechanistic discovery, cross-domain transfer learning, and scalable modeling of diverse populations. Moreover, the availability of public microbiome datasets, combined with increasingly standardized workflows, is helping to democratize access and accelerate progress in the field.

Nonetheless, challenges remain. The field must grapple with issues of data sparsity, confounding, population bias, and the interpretability of AI models. Addressing these limitations will require coordinated efforts in methodological innovation, data harmonization, benchmarking, and ethical data governance. Particular emphasis should be placed on improving model transparency, ensuring representation across global populations, and integrating privacy-preserving computational strategies for sensitive microbiome and host data.

Looking ahead, the convergence of AI with microbiome science offers profound potential—not only for improving diagnostics and precision therapeutics, but also for advancing our fundamental understanding of the microbiota–host axis. As datasets grow in size, diversity, and depth, and as AI techniques continue to mature, we anticipate a shift from retrospective association studies to real-time, predictive, and even interventional models that can inform clinical decision-making. Ultimately, AI-driven microbiome research is poised to become a cornerstone of personalized and preventive medicine in the 21st century.

By embracing these opportunities and addressing the remaining barriers, the field can move decisively toward a future where the gut microbiome is not only measured—but meaningfully interpreted, modeled, and used—to transform human health.

## Figures and Tables

**Table 1:** Representative AI Studies enabling microbiome-based therapeutic optimization (diet, probiotics, FMT, pharmacotherapy).

Study/Author (Year)	Intervention Type	AI Method Used	Model Input	Therapeutic Objective	Key Outcome
Zeevi et al. (2015) [47]	Personalized diet for glycemic control	Gradient Boosting Machine Learning	Microbiome + clinical + diet/activity logs	Predict postprandial glycemic responses and tailor diet	Personalized ML-guided diets significantly improved glycemic control vs. standard plans
Deehan et al. (2020) [48]	Defined dietary fiber structures	ML-guided metabolic modeling and correlation analysis	Metagenomics + metabolomics	Optimize SCFA output via fiber composition	Discrete fibers steered propionate and butyrate production patterns
Westfall et al. (2021) [49]	Probiotic cocktail optimization in synthetic gut (ABIOME)	Multivariate Adaptive Regression Splines (MARS)	Metabolomics of probiotic mixtures	Optimize cocktails for anti-inflammatory metabolites	Developed optimized formulations that increased SCFAs and neuroprotective compounds
McCoubrey et al. (2021) [50]	Probiotic design and drug–microbiome interaction modeling	Random Forest & Neural Networks	Microbial big data + drug metabolism databases	Predict drug–microbiome interactions for precision formulation	Identified key metabolism patterns supporting pharmacotherapy design
Wastyk et al. (2021) [56]	Fermented vs. fiber diet intervention (RCT)	Deep Learning/ML-augmented multi-omics integration	Microbiome + proteomics + cytokines	Identify diet–microbiome immune links	Fermented foods increases microbial diversity and decreasesinflammatory markers
Shtossel et al. (2023) [51]	FMT donor optimization	Random Forest + Genetic Algorithm (iMic model)	Donor 16S profiles + metadata	Predict FMT engraftment and clinical response recipient-independent	Algorithm predicted recipient richness and clinical improvement; validated in mice & humans
Kouraki et al. (2024) [57]	Omega-3 and inulin supplementation (RCT)	Elastic Net + Random Forest	Stool & serum metabolomics + microbiome	Identify metabolomic predictors of response	Discovered indolepropionate and EPA markers linked to Coprococcus shift
Murovec et al. (2024) [53]	CRC & adenoma prediction (multi-omics screening)	AutoML (Random Forest, LDA, JADBIO)	Taxonomy + genes + metabolites + pathways	Identify CRC/CRA biomarkers for early intervention	AUC ≈ 0.82 using 25 features; validated across 2951 samples
Rehman et al. (2024) [58]	Drug–microbiome interaction modeling	Graph Neural Network (GCN/GAT)	Drug structures + microbial networks	Predict drug susceptibility and optimize co-therapy	GNN achieved ≈ 93% accuracy for microbial drug susceptibility
Quinn-Bohmann et al. (2024) [52]	Personalized diet/probiotic optimization for SCFAs	Microbial Community-Scale Metabolic Modeling (MCMM) + flux balance analysis	16S & shotgun metagenomics + dietary inputs	Predict individualized SCFA production and design dietary interventions	MCMMs predicted butyrate & propionate fluxes (r ≈ 0.6–0.9); linked to clinical health markers
Björk et al. (2024) [39]	Microbiome profiling in immunotherapy (ICB)	Longitudinal Random Forest models	Serial metagenomes + clinical data	Predict response to immune checkpoint blockade	Identified microbial pathways linked to durable ICB response in melanoma
Nie et al. (2025) [55]	Global CRC burden and risk forecast	Time-series ML (ARIMA + regression)	Global Burden of Disease (1990–2021) dataset	Forecast CRC and EO-CRC trends and risk factors to 2031	Predicted rising EO-CRC rates driven by diet and BMI; supports AI-guided prevention
Pateriya et al. (2025) [54]	CRC screening and diagnostic tool (CRCpred)	Ensemble ML (XGBoost, RF, ANN + Boruta/RFE)	1728 gut metagenomes from 11 cohorts	Classify CRC vs. healthy status for precision screening	XGBoost AUC ≈ 0.91; validated on independent cohorts; public web tool for CRC risk
Sizemore et al. (2024) [36]	Infant microbiome digital twin for probiotic simulation	Temporal Machine Learning (Q-net platform)	Longitudinal microbiome + clinical data	Predict growth and neurodevelopment under probiotic intervention	Digital twin achieved ≈ 76% accuracy in forecasting growth and microbial responses

**Table 2:** Representative studies on AI-enabled personalized nutrition and microbiome-informed therapeutics.

Study/Author (Year)	Application Domain	AI Method Used	Input Data	Key Outcome/Contribution
Zeevi et al. (2015) [47]	Personalized nutrition (glycemic control)	Gradient Boosting Regression Trees	Gut microbiome, blood biomarkers, dietary logs, anthropometrics	Predicted individual postprandial glycemic responses and enabled personalized diets that significantly improved glycemic control
Korem et al. (2017) [65]	Dietary response stratification	Random Forest	Dietary intake, gut microbiome profiles, host metadata	Stratified individuals into responders and non-responders to specific macronutrient compositions
Mendes-Soares et al. (2019) [66]	Metabolic response prediction	Decision Trees, Feature Selection	Gut microbiota composition, macronutrient profiles	Validated microbiome-based glycemic prediction models across independent U.S. cohorts
Deehan et al. (2020) [48]	Precision fiber supplementation	Machine learning–guided metabolic modeling	Metagenomics, metabolomics, dietary fiber structures	Identified fiber-specific microbiome responses and optimized short-chain fatty acid production
Wastyk et al. (2021) [56]	Diet–immune modulation	ML-augmented multi-omics integration	Microbiome, proteomics, cytokines, dietary intervention data	Demonstrated microbiome-mediated immune changes in response to fermented and fiber-rich diets
Shtossel et al. (2023) [51]	Microbiome–metabolome interaction prediction	Latent variable machine learning model	Microbiome + metabolomics data	Predicted metabolite concentrations (including SCFAs) from individual microbiome composition, facilitating personalized metabolic response modeling
Björk et al. (2024) [39]	Immunotherapy response prediction	Longitudinal Random Forest models	Serial metagenomes, clinical response data	Identified microbiome features predictive of response to immune checkpoint inhibitors
Quinn-Bohmann et al. (2024) [52]	Personalized SCFA optimization	Microbial community-scale metabolic modeling + ML	16S/shotgun metagenomics, dietary inputs	Predicted individualized butyrate and propionate production to guide diet and probiotic design
Rehman et al. (2024) [58]	Drug–microbiome interaction modeling	Graph Neural Networks (GCN/GAT)	Microbial networks, drug structures, metabolic pathways	Predicted microbial drug susceptibility and optimized microbiome-aware pharmacotherapy
Nayak et al. (2024) [67]	Microbiome-informed pharmacotherapy	Deep Neural Networks	Gut microbiome profiles, drug history, host genetic variants	Predicted patient-specific responses to antidepressants and statins, supporting precision prescribing

**Table 3:** Key public gut microbiome datasets for AI research.

Dataset Name	Sample Size	Data Types	Metadata Included	AI Use Cases Enabled
Human Microbiome Project (HMP)	2000	16S, WGS, clinical metadata	Diet, BMI, disease status	Supervised learning, reference-based modeling
Integrative HMP (iHMP)	1500 (longitudinal)	WGS, metabolomics, host transcriptomics	Time series, disease-specific cohorts	Time-aware ML, multi-omics fusion, disease progression
Human Microbiome Compendium (168,000 samples)	168,000 gut-microbiome samples	16S rRNA amplicon (uniform pipeline)	Country/region, project metadata, basic sample annotation	Large-scale reference modelling, transfer learning, global diversity modelling
Human Gut Microbiome Atlas (HGMA)	Samples from 20 countries (shotgun metagenomics)	Shotgun metagenomics + species/gene pan-genomes	Country, geography, disease associations for about 23 diseases	Global species-abundance modelling, disease-association machine learning, geographic variation modelling
Longitudinal Microbiome Cohorts Collection	Multiple cohorts: e.g., 3736 samples (778 subjects) + other cohorts (114, 264, 12,276 samples)	16S, metagenome; longitudinal data	Antibiotic interventions, infant development, adult cohorts, host metadata	Time-series modelling, intervention-response prediction, dynamic microbiome modelling
MGnify	>100,000 samples	16S, WGS, protein annotations	Environmental & host metadata	Microbial diversity modeling, transfer learning
MG-RAST	>20,000 public gut sets	Raw + annotated metagenomes	Varies by project	Unsupervised learning, ecological profiling
GMrepo	58,000 gut samples	16S, WGS	Age, sex, health status	Case-control classification, microbiome signature discovery
GMrepo v2	71,642 runs/samples across 353 projects	16S + whole-genome metagenomics	Age, sex, BMI, country, antibiotic usage, 133 phenotypes	Cross-disease classification, marker discovery, multi-project feature generalization
Curated Metabolomics–Microbiome Resource	14 studies, 2900 samples	Metabolomics + 16S/WGS	Paired metabolic data	Regression models, microbial-metabolite associations
Gut Virome Database (GVD)	11,810 samples	Viral genomes & proteins	Source host, geography	Virome classification, host–virus interaction modeling
Mustard Database	6000 resistance genes	AMR determinants	Taxonomic + functional annotations	Resistome prediction, gene surveillance
Kaggle Human Gut Microbiome	3000 samples	WGS + phenotype labels	Age, gender, location	Classification challenges, educational AI training
IBD Multi’omics Database (IBDMDB; iHMP/HMP2)	~1300 longitudinal samples	Metabolomics, WGS, metatranscriptomics, proteomics	Disease activity, treatment, clinical outcomes	Longitudinal multi-omics modeling, disease progression prediction, metabolite–microbe interaction inference
Gut Microbiome–Metabolome Dataset Collection	14 studies (~2900 samples)	Metabolomics + 16S/WGS	Paired microbiome–metabolome profiles	Microbe–metabolite association learning, AI benchmarking, functional inference
MetaboLights (gut-related studies)	Thousands of studies (subset gut-focused)	Targeted & untargeted metabolomics	Study design, sample type, experimental metadata	AI-based metabolite prediction, cross-study metabolomics modeling
Metabolomics Workbench/MetabolomeXchange	>2000 public metabolomics studies	Metabolomics (LC–MS, GC–MS, NMR)	Clinical, dietary, experimental metadata	AI training/validation for metabolite profiling and functional phenotype modeling

**Table 4:** Summary of cutting-edge techniques for AI in gut microbiome research.

Technique	Key Application	Unique Advantages	Ref.
Transformer Language Models	Microbial feature representation, disease prediction	Captures taxon context and ecological semantics; improves generalization	[21,88,89]
Sequence-based Transformers	Protein/Gene function prediction	Learns from millions of sequences; supports cross-species annotation	[92,95]
Graph Neural Networks (GNNs)	Phylogeny-aware prediction, microbial networks	Utilizes structure (co-occurrence, phylogeny); models community-level dependencies	[58,96,97]
Multi-modal Deep Learning	Integrated microbiome–host phenotype prediction	Leverages synergy across omics, diet, imaging; boosts model accuracy and interpretability	[98,99,100]
Multi-omics Integration Frameworks	Systems-level disease modules, causal inference	Links microbes and metabolites; reveals joint disease signatures	[78,100,103]
Generative AI (VAEs, Diffusion Models)	Imputation, augmentation, domain transfer	Denoises sparse data; generates realistic synthetic samples	[90,104]
Causal Inference & Explainable AI	Mechanistic insight and feature attribution	Improves interpretability; links microbial drivers to host outcomes	[105,106]
Federated & Privacy-Preserving Learning	Cross-center model training without data sharing	Enables global collaboration; preserves data confidentiality and equity	[107,108]
Few-Shot and Transfer Learning	Adaptation to rare taxa or small cohorts	Boosts generalization in low-sample microbiome studies; reuses pretrained embeddings	[90,109]
Temporal & Digital Twin Models	Longitudinal microbiome simulation, intervention forecasting	Captures dynamic host–microbe co-evolution; supports in silico therapeutic testing	[33,36]
AutoML and Hybrid AI Frameworks	Automated model selection for complex microbiome data	Streamlines multi-omics workflows; optimizes performance with minimal manual tuning	[53,54,100]

## Data Availability

No new data were created or analyzed in this study. Data sharing is not applicable to this article.

## Abbreviations

The following abbreviations are used in this manuscript:

AI	Artificial intelligence
ML	Machine learning
DL	Deep learning
FMT	Fecal microbiota transplantation
IBD	Inflammatory bowel disease
SCFA	Short-chain fatty acid
GEM	Genome-scale metabolic model
COBRA	Constraint-based reconstruction and analysis
HMP	Human Microbiome Project
iHMP	Integrative HMP
GVD	Gut virome database
GNN	Graph neural network
VAE	Variational autoencoder
